# Lung progenitors from lambs can differentiate into specialized alveolar or bronchiolar epithelial cells

**DOI:** 10.1186/1746-6148-9-224

**Published:** 2013-11-08

**Authors:** Fabienne Archer, Alain Abi-Rizk, Sophie Desloire, Christine Dolmazon, Barbara Gineys, François Guiguen, Vincent Cottin, Jean-François Mornex, Caroline Leroux

**Affiliations:** 1 Université de Lyon, Lyon F-69007, France; 2 Université Lyon 1, Lyon F-69007, France; 3 INRA, UMR754, "Rétrovirus et Pathologie Comparée", UMS 3444 SFR BioSciences Gerland - Lyon Sud, Lyon F-69007, France; 4 Hospices Civils de Lyon, Lyon F-69007, France

**Keywords:** Lung, Progenitor, Bronchioloalveolar, SP-C, CCSP, CD34, Sheep, Differentiation, Maintenance

## Abstract

**Background:**

Airways progenitors may be involved in embryogenesis and lung repair. The characterization of these important populations may enable development of new therapeutics to treat acute or chronic lung disease. In this study, we aimed to establish the presence of bronchioloalveolar progenitors in ovine lungs and to characterize their potential to differentiate into specialized cells.

**Results:**

Lung cells were studied using immunohistochemistry on frozen sections of the lung. Immunocytochemistry and flow cytometry were conducted on *ex-vivo* derived pulmonary cells. The bronchioloalveolar progenitors were identified by their co-expression of CCSP, SP-C and CD34. A minor population of CD34^pos^/SP-C^pos^/CCSP^pos^ cells (0.33% ± 0.31) was present *ex vivo* in cell suspensions from dissociated lungs. Using CD34 magnetic positive-cell sorting, undifferentiated SP-C^pos^/CCSP^pos^ cells were purified (>80%) and maintained in culture. Using synthetic media and various extracellular matrices, SP-C^pos^/CCSP^pos^ cells differentiated into either club cells (formerly named Clara cells) or alveolar epithelial type-II cells. Furthermore, these *ex vivo* and *in vitro* derived bronchioloalveolar progenitors expressed *NANOG*, *OCT4* and *BMI1*, specifically described in progenitors or stem cells, and during lung development.

**Conclusions:**

We report for the first time in a large animal the existence of bronchioloalveolar progenitors with dual differentiation potential and the expression of specialized genes. These newly described cell population in sheep could be implicated in regeneration of the lung following lesions or in development of diseases such as cancers.

## Background

It is now established that post-natal and adult lungs contain multiple progenitors or stem cell populations able to participate to organ homeostasis and repair [1]. The relative distribution of these populations varies between species [2,3] and these cells are difficult to evidence as the consequence of the low turn-over of the respiratory epithelium [4]. The mouse system allowing the study of cell regeneration in response to chemical injury and more recently *in vivo* genetic lineage tracing [5,6] has supplied a model to explore and describe epithelial lineages implicated in lung repair. Even so the murine pulmonary epithelium is rather different from the ones of the large mammals. In small mammals such as rodents, the proximal airways are composed of a layer comprising one or two cells thick that rests on a very sparse network of basal cells [7-10]. In large mammals, this epithelium is characterized by pseudo-stratified columnar cells together with ciliated, secretory, and parabasal cells linked to a foundation of basal cells [11,12]. Moreover club cells, formerly named Clara cells [13-15] are present throughout the mouse airways while confined to the most distal bronchiolar airways in humans. Regeneration of alveolar tissue is rapid and efficient in rodents, but takes longer and needs higher level of stimulation in large mammals [16]. Taking in account these observed differences on composition and temporal dynamics, the description of the resident progenitor populations is of importance in large mammals to better understand the building, maintenance and repair of the lung epithelium. Moreover, sheep have a long history as an experimental model to study respiratory diseases. These small ruminants have been proposed as good candidates for vaccine development as well as for comprehensive studies on asthma, bronchial obstruction or infant respiratory distress syndrome. It also provides a very useful model for respiratory infections and lung cancer.

Cystic fibrosis, chronic obstructive pulmonary disease and lung adenocarcinoma are part of the lung diseases that affect the distal lung and could appeal for progenitor or stem cell activation. The bronchioloalveolar region has been extensively studied in normal and injured lungs of rats and mice in response to chemical treatments. Undifferentiated cells in the bronchioloalveolar duct junction have been histologically identified as different from the alveolar epithelial type II cells (AECII), the club cells (Clara) or the ciliated cells [17]. In mice, these bronchioloalveolar progenitors, referred as BASCs (BronchioloAlveolar Stem Cells), share phenotypic characteristics of both club cells and AECIIs, suggesting a niche of progenitors [2,17-20]. They express CCSP (Club Cell Secretory Protein) specific of the club cells, SP-C (Surfactant Protein C), a component of the pulmonary surfactant produced by the AECII as well as CD34, a surface antigen of the hematopoietic stem cells [18,21]. Murine BASCs appear to be resistant to bronchiolar and alveolar damage *in vivo*, proliferate during epithelial repair and have the potential to give rise to club cells and AECIs or AECIIs [18,21]. Whether analogous pulmonary epithelial progenitors are present and could differentiate into specialized bronchiolar or alveolar cells in large animals is currently unknown.

We looked for bronchioloalveolar progenitors in sheep by *ex vivo* isolation of CD34^pos^/SP-C^pos^/CCSP^pos^ cells. Using CD34-magnetic positive cell selection, we isolated a SP-C^pos^/CCSP^pos^ viable cell population from the lungs of 0 to 3 month old lambs. Synthetic media and various extracellular matrix were used to establish the *in vitro* conditions to maintain SP-C^pos^/CCSP^pos^ cells in an undifferentiated and proliferative state or, alternatively, to induce their differentiation into either club cells or AECIIs. The bronchioloalveolar progenitors obtained *ex vivo* or maintained *in vitro* were further shown to express genes involved in stem cells or lung development such as *NANOG* (Nanog homeobox), *OCT4* (Octamer-binding transcription factor 4) and *BMI1* (polycomb ring finger oncogene). The expression of these genes was modulated upon exposure to culture conditions favoring cell differentiation.

## Results

### *In vivo* description of SP-C^pos^, CCSP^pos^ and SP-C^pos^/CCSP^pos^ pulmonary cells

**Figure 1:**
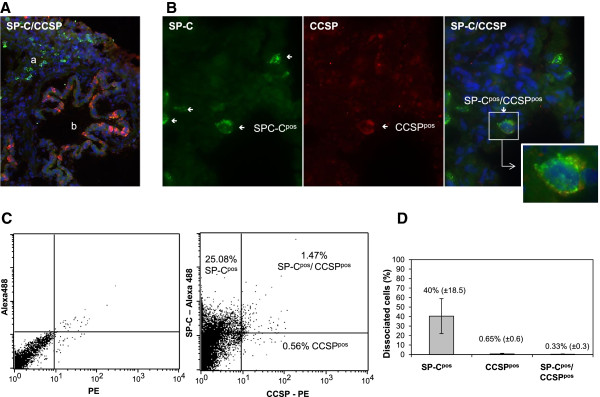
***In vivo *localization of SP-C**^**pos**^**/CCSP**^**pos **^**cells.** Frozen lung sections from ten 0 to 3 month old lambs were analyzed by immunohistochemistry for the expression of SP-C (green), CCSP (red) and the co-expression of SP-C and CCSP. The nuclei were stained with DAPI (blue). **(A)** Lung cross sections showed the expression of CCSP in the bronchiole (b) and SP-C in the alveoli (a) (100x magnification). **(B)** Immunostaining of rare SP-C^pos^/CCSP^pos^ cells *in situ* (400x magnification). Inset: enlargement of a SP-C/CCSP double-positive cell. **(C)** SP-C and CCSP expression from a representative lamb (# 1507) in *ex vivo* dissociated cells by flow cytometry showing the presence of a SP-C^pos^/CCSP^pos^ population. **(D)** Frequency of SP-C^pos^, CCSP^pos^ and SP-C^pos^/CCSP^pos^ cells from ten 0 to 3 month old lambs. The data are expressed as the mean (± SD) percentage of the cells expressing the cellular markers.

The expression of SP-C and CCSP was analyzed in the lungs of 0 to 3 month old lambs by immunohistochemistry on frozen sections using cross-reacting antibodies. As expected, these antibodies specifically recognized cells expressing SP-C in the alveoli and cells expressing CCSP in the bronchioli (Figure 1A) validating their use as specific markers of respectively AECIIs and club cells in sheep. AECIIs were easily detectable in most of the sections due to their high expression of SP-C (Figure 1B) and club cells expressing CCSP were detectable when bronchioli were present on the lung section. Interestingly, rare SP-C^pos^/CCSP^pos^ cells were observed in some lung sections (Figure 1B), demonstrating the presence of bronchioloalveolar progenitors in newborn lambs. Among the 4 independent lungs and repeated immuno-stainings, the SP-C^pos^/CCSP^pos^ cells remained rare and only present on few lung sections. But this first *in vivo* evidence for the presence of progenitor-like pulmonary cells prompted us to search for these rare cells among cells obtained from lung dissociation.

To quantify the respective frequency of AECIIs, club cells and progenitors, cell suspensions obtained by enzymatic dissociation of lung tissue from 10 lambs (0 to 3 month old) were analyzed for their expression of SP-C and CCSP by flow cytometry. Based on the dot plot analysis from a representative lamb (Figure 1C) and the compiled data from 10 lambs (Figure 1D), the suspensions of lung cells repeatedly contained 40% (± 18.5) SP-C^pos^ cells and 0.65% (± 0.60) CCSP^pos^ cells (Figure 1D). Co-expression of SP-C/CCSP was then analyzed to quantify the bronchioloalveolar progenitors. An average of 0.33% (± 0.31) of the total cells co-expressed the two markers and presented an immunophenotype compatible with lung progenitors (Figure 1D). We noted the low percentage of club cells in the cell suspensions issued from tissue dissociation. This may be attributed to our tissue processing. In order to work mainly with the cell populations present into the lung parenchyma, we carefully have macro-dissected the lung tissues before enzymatic digestion in order to limit cells coming from the bronchi and bronchioli. Moreover, the number of club cells in the lung of large mammals is lower compared to rodents.

### *In vitro* expansion and phenotypic characterization of primary airway epithelial cells

**Figure 2:**
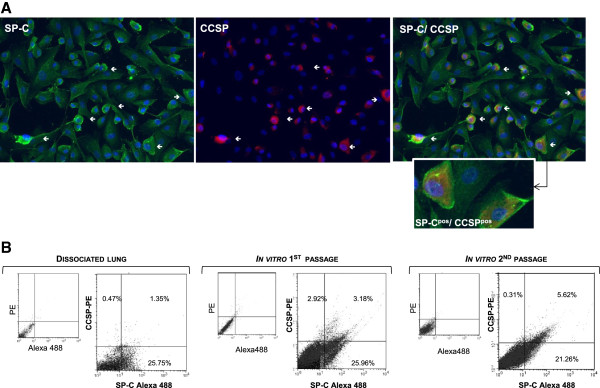
***In vitro *expansion of SP-C**^**pos**^**/CCSP**^**pos **^**progenitors.** Pulmonary epithelial cells from dissociated lung tissues were cultured *in vitro* on fibronectin-coated plates supplemented with "complete Q286 medium". **(A)** Lung epithelial cells derived from lamb #1487 and maintained for 2 passages were analyzed by immunostaining for the expression of SP-C (green), CCSP (red) and the co-expression of SP-C and CCSP (400x magnification). Inset: enlargement of double-positive cells. **(B)** Flow cytometry analysis of a representative animal shows the percentage of SP-C^pos^ cells and CCSP^pos^ cells above the background fluorescence. The double staining shows an enriched culture of AECIIs with the presence of SP-C^pos^/CCSP^pos^ bronchioloalveolar progenitors after dissociation, and at the first and second passage *in vitro*. For each condition, the left graph (smaller) corresponds to controls and the right graph (larger) presents SP-C and CCSP specific immunostaining.

Dissociated cells were grown in culture with selective Quantum286 synthetic medium complemented with KGF and HGF (referred to "complete Q286 medium") on fibronectin-coated plates and maintained for two to three passages as previously described [22]. Small and large cubical epithelial cell colonies and a few spindle-shaped cells were rapidly observed after 3 days of culture; the small cubical cells proliferated and were further characterized for the expression of SP-C and CCSP. As shown by specific labeling in immunocytochemistry and flow cytometry, a majority of the observed cubical cells were SP-C^pos^ AECIIs, while few of them were CCSP^pos^ club cells (Figure 2A and 2B). Interestingly, a minor population of SP-C^pos^/CCSP^pos^ cells, compatible with bronchioloalveolar progenitors, was reproducibly identified in culture (Figure 2A and 2B). Compared to the AECIIs, the SP-C^pos^/CCSP^pos^ cells were semi-adherent round cells with small cytoplasmic extensions (Figure 2A). These double positive SP-C^pos^/CCSP^pos^ cells were rare after lung dissociation but enriched and maintained upon *in vitro* culture with synthetic medium (Figure 2B).

### CD34 selection of bronchioloalveolar progenitors

**Figure 3:**
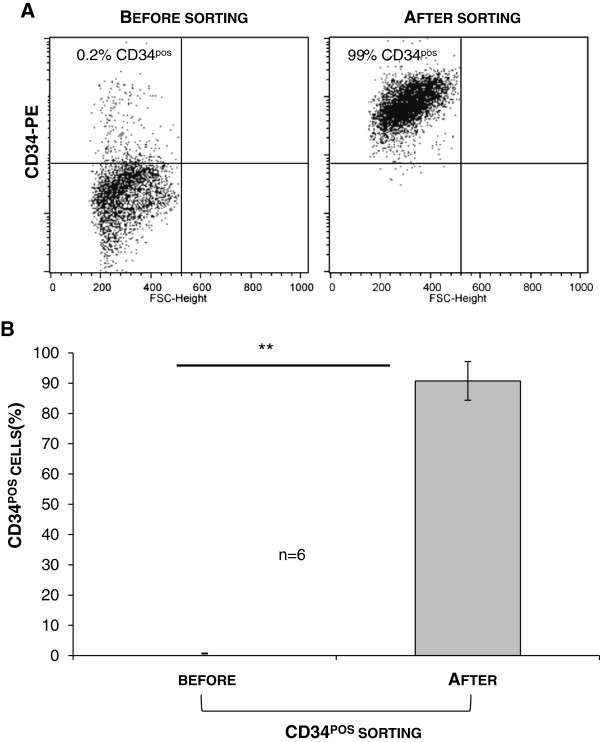
**Enrichment in CD34**^pos ^**cells by magnetic cell sorting.** Lung primary cells from lamb lungs were subjected to magnetic cell sorting with anti-ovine CD34, then analyzed by flow cytometry. **A**: Representative dot plot from one animal showing expression of CD34^pos^ cells in the dissociated tissue (Before sorting) and after the positive selection (After sorting) **B**: Enrichment of CD34^pos^ cells from independent magnetic cell sorting from 6 lambs shown as the mean (± SD). **: p < 0.01.

To isolate and enrich a viable population of bronchioloalveolar progenitors, cells from *ex vivo* dissociated lungs were positively selected using anti-CD34 antibody-coated magnetic beads without any prior cell culture step. Cell surface expression of CD34, a surface protein expressed by some progenitors or stem cells, was then measured by flow cytometry to assess the enrichment of the bronchioloalveolar progenitor population in the selected population. In the 6 tested lambs, the CD34 cell sorting remarkably enriched the CD34^pos^ population from 0.7% (± 0.12) after tissue dissociation to over 90% in the CD34-selected single cell suspension (Figure 3) with greater than 85% viability, as measured by trypan blue exclusion.

### Expression of SP-C and CCSP among the CD34^pos^-selected population

**Figure 4:**
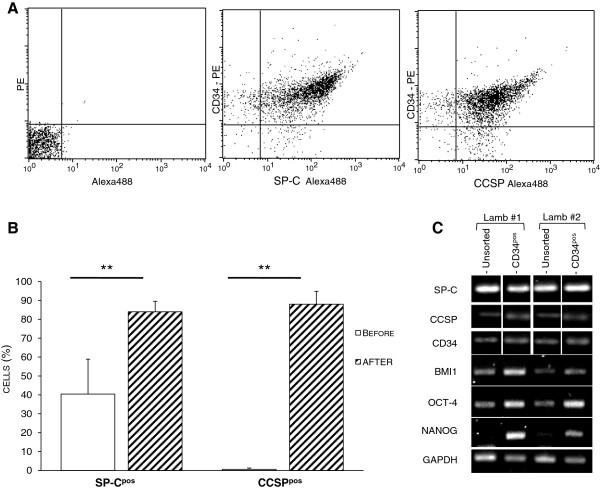
**Expression of SP-C and CCSP in CD34**^**pos**^** cells. (A)** Dot blot analysis from a representative animal (lamb #1744) showing the expression of SP-C and CCSP in the majority (>90%) of the CD34^pos^ cells. **(B)** Combined results from 10 lambs showing the enrichment of SP-C^pos^/CCSP^pos^ cells in the CD34-selected cell fraction. The data are shown as the mean (± SD). **: p < 0.01. Before: before CD34 sorting; After: after CD34 sorting. **(C)** Expression of *SP-C*, *CCSP*, *CD34*, *BMI1*, *OCT4*, *NANOG* and *GAPDH* mRNAs in the unselected and in the CD34-selected cells measured by RT-PCR.

To quantify the bronchioloalveolar progenitors among the CD34^pos^ population, the selected cells were analyzed for the expression of CCSP and SP-C. By flow cytometry, the majority (over 80%) of the CD34^pos^ cells co-expressed CCSP and SP-C with respectively 88% (± 6.8) and 84% (± 5.5) positive cells (Figure 4A and 4B). We assumed that hematopoietic stem cells or endothelial progenitors may contribute to the SP-C^neg^ and CCSP^neg^ cells among the CD34^pos^ population. Due to the nature of the antibodies used (i.e., polyclonal rabbit anti-proSP-C, mouse monoclonal or polyclonal rabbit anti-CCSP and monoclonal mouse anti-CD34), direct double labeling of SP-C and CCSP on the CD34^pos^ population was not technically achievable. However, the large proportion of SP-C^pos^/CD34^pos^ and CCSP^pos^/CD34^pos^ cells clearly demonstrated that most of the CD34^pos^ cells were SP-C^pos^/CCSP^pos^ progenitors. The proportion of SP-C^pos^/CCSP^pos^ cells among the CD34^pos^ population was similar in the 6 lambs analyzed independently (Figure 4B). Importantly, the proportion of CD34^neg^/SP-C^pos^ and CD34^neg^/CCSP^pos^ cells was very low to undetectable in the CD34-sorted cell suspension (1.0% ± 1.7 and 1.1% ± 1.8, respectively), confirming that the procedure efficiently purified CD34^pos^ cells and not AECIIs or club cells. Taken together, our results suggested that a bronchioloalveolar progenitor population exists in lambs and that these cells concomitantly express CD34, SP-C and CCSP.

To further characterize the CD34^pos^ selected population, the mRNA expression of genes known to be preferentially but not exclusively expressed in stem cells, such as the self-renewal factor gene *BMI1* and the pluripotent state-specific transcription factor genes *OCT4* and *NANOG*, were analyzed. The expression of the housekeeping gene *GAPDH* was analyzed as a reference gene. Compared to the total cell suspensions obtained after lung dissociation, the CD34^pos^ population expressed higher levels of *BMI1*, *OCT*4 and *NANOG*, suggesting the enrichment of progenitors during the purification process (Figure 4C). Interestingly, *NANOG* was almost absent from the unsorted population but was strongly expressed in the CD34^pos^-sorted cells (Figure 4C). Importantly, the expression of SP-C, CCSP and CD34 was maintained after CD34 selection (Figure 4C). Unfortunately the low number of cells obtained during the selection process did not allow us to further quantify the level of gene expression, due to the very low amounts of total RNA per analyzed sample.

### *In vitro* maintenance of the ovine bronchioloalveolar progenitors

**Figure 5:**
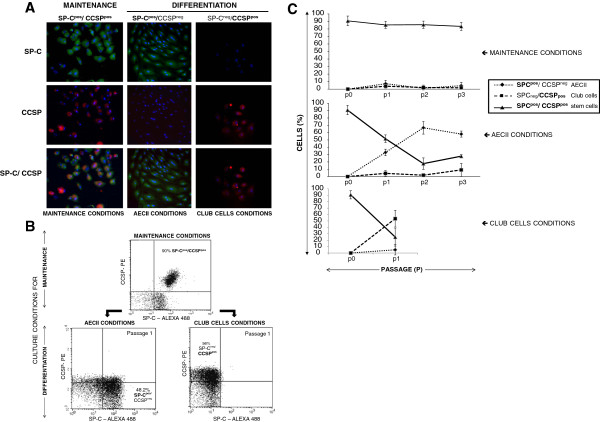
**In vitro maintenance and differentiation of bronchioloalveolar progenitors.** CD34^pos^/SP-C^pos^/CCSP^pos^ cells from explanted lungs were maintained either in a. “maintenance conditions” with "complete Q286 medium" on fibronectin and collagen-coated inserts, b. differentiated in “AECII conditions” with "complete Q286 medium" on fibronectin and collagen-coated plates or c. in “Club conditions” with "basic Q286 medium" on fibronectin-coated plates. **(A)** Representative immunostaining from cells isolated from one lamb for SP-C (green) and CCSP (red). Co-expression was performed on *in vitro* maintained bronchioloalveolar progenitors (SP-C^pos^/CCSP^pos^) or differentiated AECIIs (SP-C^pos^/CCSP^neg^) or club cells (SP-C^neg^/CCSP^pos^). **(B)** The population of CD34^pos^/SP-C^pos^/CCSP^pos^ cells from a representative lamb grown in maintenance or differentiation media was quantified by flow cytometry for the expression of SP-C or CCSP. The results are presented as dot plots of the expression of both markers. **(C)** CD34^pos^/SP-C^pos^/CCSP^pos^ cells from 6 independent lambs cultured in maintenance or differentiation media were followed over three passages (p0 to p3) by flow cytometry for the expression of SP-C and CCSP. The results are presented as the mean ± SD of the cells expressing each phenotype.

To assess their ability to proliferate *in vitro*, selected CD34^pos^ progenitors were seeded onto inserts (membrane with 0.4 μm pore size) coated with fibronectin and type I and IV collagens and cultured with "complete Q286 medium" under “maintenance conditions" (Figure 5A, 5B and 5C). After 15 days, these CD34^pos^ cells gave rise to small colonies of cubical epithelial cells, most of which expressed SP-C and CCSP (Figure 5A). The majority of these cells (>90%) maintained the co-expression of SP-C and CCSP for up to 3 passages (corresponding to approximately 45 days in culture) (Figure 5B and 5C). When maintenance conditions were applied to cells from 6 independent animals, the same proportions of SP-C^pos^/CCSP^pos^ cells were generated, and this population of bronchiololaveolar progenitors was maintained over time (Figure 5C). While SP-C and CCSP expression persisted, the overall fluorescence intensity of these two markers decreased over time to reach a lower but easily detectable signal after 3 passages.

### From bronchioloalveolar progenitors to differentiated epithelial pulmonary cells *in vitro*

In addition to their self-renewing properties, progenitors must be able to differentiate into multiple cell types under specific conditions. Indeed, using synthetic medium and adapted cell culture conditions, CD34^pos^/SP-C^pos^/CCSP^pos^ bronchioloalveolar progenitors had the potential to differentiate into either AECIIs or club cells. To induce the differentiation of SP-C^pos^/CCSP^pos^ cells into AECIIs, CD34^pos^ cells were seeded onto plates coated with fibronectin and type I and IV collagens and cultured with "complete Q286 medium", culture conditions that we defined as "AECII conditions". Along 3 passages (approximately 45 days), we observed a noticeable increase of the proportion of cell expressing SP-C^pos^/CCSP^neg^ (Figure 5A and B), whereas the SP-C^pos^/CCSP^pos^ population decreased over time (Figure 5C). Cultures from 6 independent animals showed the same modulation with an increasing proportion of SP-C^pos^/CCSP^neg^ AECIIs (from < 5% to 58%; Figure 5C). In contrast, the population of SP-C^pos^/CCSP^pos^ progenitors rapidly decreased throughout the passages from over 90% to less than 30% (Figure 5C). Under these conditions, no CCSP^pos^ cells emerged from the initial SP-C^pos^/CCSP^pos^ population. Because we have previously shown that none to less than 1% (± 1.7) of the CD34^neg^/SP-C^pos^ AECII were present after CD34 positive selection, our findings strongly argue for the *in vitro* differentiation of bronchioloalveolar progenitors into AECIIs and not the mere proliferation of pre-existing AECIIs.

Conversely, CD34^pos^ progenitors cultured in "basic Q286 medium" (without KGF and HGF) on fibronectin-coated plates, conditions that we defined as "club cell conditions" gave rise to colonies containing primarily SP-C^neg^/CCSP^pos^ cells (53.6% ± 13) after 15 days in culture (Figure 5A, 5B and 5C). Concurrently, SP-C^pos^/CCSP^pos^ bronchioloalveolar progenitors (24.9% ± 14) rapidly decreased, while SP-C^pos^/CCSP^neg^ AECIIs remained low to undetectable (5% ± 8; Figure 5C). Similarly to the AECII conditions, we have previously shown that none to less than 1.1 (± 1.8%) of the CD34^neg^/CCSP^pos^ club cells were present after CD34 positive selection from the 6 tested lambs, strongly suggesting the *in vitro* differentiation of bronchioloalveolar progenitors into club cells. Together with the low proliferation rate of CCSP^pos^ cells in the cultures, our findings strongly argue for the differentiation of bronchioloalveolar progenitors into club cells rather than the proliferation of already existing club cells in the cultures.

### Expression of *NANOG*, *OCT4* and *BMI1*

**Figure 6:**
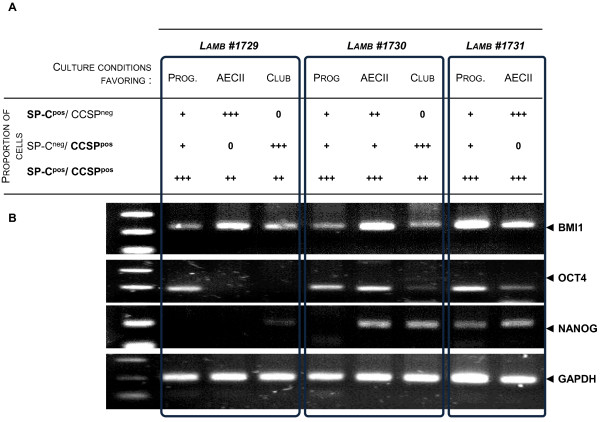
***BMI1 *and *OCT4 *mRNA expression in maintained and differentiated CD34**^**pos**^**/SP-C**^**pos**^**/CCSP**^**pos **^**cells. (A)** Depending on the culture medium conditions (maintenance, AECII or club medium), the relative proportions of SP-C^pos^/CCSP^pos^, SP-C^pos^/CCSP^neg^ or SP-C^neg^/CCSP^pos^ were determined at the first passage by flow cytometry and reported in the table as the following: 0% for no cells, + for <15%, ++ ≥15% and ≤50%, and +++ for ≥50%. **(B)** The *BMI1*, OCT4 and *NANOG* mRNAs were detected by RT-PCR from the total cell RNAs extracted from the three culture conditions at passage 1 and compared to the ovine *GAPDH* housekeeping gene. Prog: progenitors.

As previously mentioned, the expression of *NANOG* and *OCT4* is a strong indicator of a stem cell/ progenitor phenotype. *NANOG* and *OCT4* were expressed in the CD34^pos^/SP-C^pos^/CCSP^pos^ cells enriched from dissociated tissues (Figure 4C). To further characterize the CD34^pos^ cells that were maintained *in vitro* and differentiated, RT-PCR was used to analyze the expression of *OCT4*, *NANOG* and *BMI1* in the progenitors maintained in culture and in the different enriched, but not pure, cultures of AECIIs and club cells derived from 3 lambs (#1729, #1730, #1731) at passage 1 (Figure 6A). *BMI1* was expressed at various levels and predominantly in the medium that favored the differentiation of bronchioloalveolar progenitors into AECIIs (Figure 6B). Importantly, this first passage still contained SP-C^pos^/CCSP^pos^ progenitors. Interestingly, *OCT4* was expressed in the “maintenance conditions” that favored expansion of bronchioloalveolar progenitors, in which greater than 70% of the cells displayed a SP-C^pos^/CCSP^pos^ phenotype. In the differentiation conditions that generated AECIIs or club cells, *OCT4* expression was strongly reduced in 2 of the 3 lambs. However, in lamb #1730, some expression of *OCT4* was detectable in cells cultured in “AECII conditions”, which is compatible with the lower proportion of SP-C^pos^/CCSP^neg^ AECIIs compared to the 2 others lambs at passage 1 (Figure 6A). This result suggests that *OCT-4* expression was characteristic of cell progenitors and that its expression was lost upon differentiation. Surprisingly, the expression of *NANOG* appeared to be less stable in the bronchioloalveolar progenitors maintained *in vitro* (Figure 6) compared to the ones obtained *ex vivo*, as previously described (Figure 4C), suggesting that the cultured SP-C^pos^/CCSP^pos^ cells rapidly underwent a differentiation step.

## Discussion

In this study, we described the presence of SP-C^pos^/CCSP^pos^ progenitors in the lung parenchyma of 0-3 month-old lambs. These rare cells (~0.3% of the pulmonary epithelial cells) have been isolated after tissue dissociation and they co-expressed CD34, SP-C and CCSP. The SP-C^pos^/CCSP^pos^ cells were maintained *in vitro* for up to 2 or 3 passages (30-45 days). Pluripotent progenitors are defined by their ability to maintain and differentiate upon exposure to specific stimuli. To further characterize the ovine SP-C^pos^/CCSP^pos^ cells, we first performed positive selection for CD34 expression, a surface protein widely described on various stem cells and present on ovine hematopoietic stem/progenitor cells [23]. A small population of cells (few thousand) was isolated and characterized as SP-C^pos^/CCSP^pos^ cells. They expressed mRNAs for the *NANOG*, *OCT4* and *BMI1* genes which are molecular markers of stem cells/progenitors. They retained the expression of *SP*-C, *CCSP* and *CD34* mRNAs over time. Importantly, the CD34^pos^/SP-C^pos^/CCSP^pos^ cells were able to maintain for up to 3 passages *in vitro*. The CD34^pos^/SP-C^pos^/CCSP^pos^ cell population retained the potential to differentiate at least into AECIIs or club cells in specific culture conditions. The CD34^pos^/SP-C^pos^/CCSP^pos^ cells, maintained in *vitro*, expressed *OCT4* and *BMI1* mRNAs, conferring them characteristics inherent to multipotent cells or progenitors.

Bronchioloalveolar progenitors have been reported in mice in an anatomically well-defined location at the bronchioloalveolar junction where conducting and respiratory airways meet [18,24,25]. These cells were initially described by their expression of Sca-1 and CD34, and their co-expression of SP-C and CCSP. We succeeded in demonstrating the *in vivo* presence of rare ovine bronchioloalveolar progenitors in the lungs of newborn or young animals (less than 3 months old), while other groups have difficulties to identify bronchioloalveolar progenitors in sheep upon searching for these cells using CCSP and SP-C immunostaining on lung sections [26,27]. Interestingly, two studies have reported rare to extremely rare SP-C^pos^/CCSP^pos^ cells in virally-induced lung tumors in sheep [26,28]. Taken together, these results are consistent with the presence of SP-C^pos^/CCSP^pos^ cell population, extremely rare *in vivo* on frozen lung sections and representing a new progenitor population. The discrepancy between our study and previous studies [26,27] may be explained by the low but clearly above background levels of SP-C and CCSP expression in double-positive cells compared to the expression of these markers in AECIIs and club cells, respectively. Moreover, a recent study on ovine lung sections, have shown that CCSP^pos^ cells were not (preterm lung) or poorly (9 days old lung) detectable in prenatal and early postnatal lung compared to mature lung (91 days) in bronchi, terminal bronchioles and respiratory bronchiole [29]. These elements point the influence of the age of the lung studied, in the search for postnatal bronchioloalveolar CCSP^pos^/SP-C^pos^ progenitors.

The visualization of double-positive cells in the complex lung is therefore challenging and the CD34 enrichment strategy enabled us to evidence and to study this progenitor population. In addition, bronchioloalveolar progenitors may be more easily identified in lungs from newborn lambs. Of note, we have previously reported the presence of SP-C^pos^/CCSP^pos^ cells in the sheep lung while describing the *ex vivo* isolation of tumoral AECIIs from a virus-induced lepidic lung adenocarcinoma [22]. While culturing these cells in three-dimensional conditions, better adapted to these polarized and specialized cells, we repeatedly observed SP-C^pos^/CCSP^pos^ double-positive cells (data not shown). These results prompted us to go further into their characterization.

SP-C^pos^/CCSP^pos^ double-positive bronchioloalveolar progenitors are rare and represent less than 0.4% of the total epithelial cells from dissociated lungs. In comparison, a recent study has established that undifferentiated human stem cells identified by c-kit immunosorting are present at a frequency of 1 per 6,000 cells in the bronchioles and 1 per 30,000 cells in the alveoli in adults [30]. In fetal human lung tissue at 12-36 weeks of gestation, the frequency of stem cells varies from 1 per 11,000 to 1 per 600 cells, with an average of 1 per 4,100 cells [30]. Our CD34-positive selection procedure was crucial to isolate bronchioloalveolar progenitors from the lung even though the absolute number of CD34^pos^/SP-C^pos^/CCSP^pos^ cells was ultimately low (only a few thousands cells) and confirmed the existence of a rare population of SP-C^pos^/CCSP^pos^ bronchioloalveolar progenitors. Importantly, all of the selection steps were performed *ex vivo*, without any cell culture steps and in the absence of fetal calf serum known to induce cell differentiation.

The CD34^pos^/SP-C^pos^/CCSP^pos^ population resembles the now well-characterized murine bronchioloalveolar stem cells in terms of both morphology and cell markers [18,24,25]. We clearly demonstrated that ovine bronchioloalveolar progenitors behave as multipotent precursors in culture and have maintenance and differentiation potential upon modulation of the culture conditions.

We then demonstrated that the *ex vivo*-derived ovine bronchioloalveolar progenitors retained the expression of the *BMI1*, *OCT4* and *NANOG* genes which are some of the most important markers of undifferentiated pluripotent cells and are major players in embryonic and adult stem cells. BMI1 is a member of the PcG family of transcription repressors that play crucial roles in development, stem cell biology and cancer [31]. *BMI1* is also highly expressed in tumors and regulates the cell fate of cancer cells and normal and tumoral stem cells [32,33]. *BMI1* participates in the maintenance of endogenous stem cells, partly by repressing genes involved in cell death and senescence [34]. In mice, BMI1 is necessary for bronchioloalveolar stem cells expansion [35]. Similarly to murine bronchioloalveolar stem cells, ovine bronchioloalveolar progenitors expressed *BMI1* under maintenance/self-renewing conditions, and this expression was not modified by exposure to differentiation conditions.

While OCT4, a member of the POU (Pit-Oct-Unc) transcription factor family, is essential for the maintenance of self-renewal capacities, NANOG (a downstream target of OCT4) contributes to the cell fate determination of pluripotent cells during embryogenesis [36,37]. OCT4 and NANOG are among the few key factors that enable the reprogramming of adult somatic cells into pluripotent stem cells [38-42]. Interestingly, induced pluripotent cells (iPSCs) generated from sheep fibroblasts have recently been demonstrated to exhibit an embryonic stem cell-like morphology and to express OCT4 and NANOG among other intracellular and surface markers associated with undifferentiated cells, as previously demonstrated in humans and mice [43]. The level of OCT4 is critical for the state of a cell and its inactivation results in the loss of pluripotency and induces differentiation [44]. We could observe a loss or reduction of *OCT4* gene expression in AECII and club cells compared to progenitors. OCT4 and NANOG are expressed in lung stem cells, and their co-expression may enhance malignancy by inducing cancer stem cell-like properties [45,46]. Surprisingly, NANOG gene expression was higher in AECII and club cells than in progenitors in our *in vitro* culture conditions. NANOG expression is known to be regulated by a number of pluripotent transcription factors (FoxD3, SOX2, OCT4) [47]. It is likely that this complex regulation is also dependent of the *in vitro* context (growth factors, monolayer or 2D culture conditions) and others active signaling cascades [48]. With respect to the recently described human c-kit lung stem cell [30], it would be interesting to study the expression of c-kit in lamb lung sections and in the SP-C^pos^/CCSP^pos^ bronchioloalveolar progenitors population to better understand the hierarchical organization of these progenitors in this complex tissue.

## Conclusions

We report here the first direct characterization of CD34^pos^/SP-C^pos^/CCSP^pos^ bronchioloalveolar progenitors from the lung of a large animal. While extremely rare, these cells can be purified and enriched from the lungs of newborn lambs. These cells have a potential to maintain and differentiate in highly specialized epithelial cells. The SP-C^pos^/CCSP^pos^ bronchioloalveolar progenitors express progenitor markers, such as the *NANOG*, *BMI1* and *OCT4* genes, *in vivo* and *in vitro*, indicating that newborn lung progenitors retain characteristics typical of progenitors residing in the developing organ. Although dealing with a limited number of cells, these data enable the study of the development of the bronchiolar and alveolar epithelia. Sheep, an alternative animal model closer to humans than mice, are already largely used to study lung physiology and pathology. In this context, ovine bronchioloalveolar progenitors may represent new tools to study lung regeneration or new therapeutic targets.

## Methods

### Animals

Lungs were collected from 10 young lambs either from 3 month old animals at the Corbas slaughterhouse (Corbas, Rhône, France), with the formal authorization for the access to the facility and under the supervision of Dr F. Guiguen (DVM) or from stillborn lambs from healthy ewes belonging to the flock providing lambs and sheep to the experimental infectious disease platform of the INRA Animal Health Division, Tours France; these lungs have been sampled by qualified staff and DVM of the core facility. None of the animals used in this study were engaged into an experimental protocol.

Immediately after death, fractions of the lungs were processed as previously described [22]. Lungs were minced into small pieces and incubated in 10 μg/ml of DNAse I, 1 mg/ml protease XIV and 0.025% collagenase Ia for 2 h at 37°C, filtered through 100 μm and 40 μm cell strainers and centrifuged at 450 g for 10 min at 4°C. The cell pellets were collected in a red blood cell lysis buffer (0.15 M NH_4_Cl, 10 mM KHCO_3_, 0.1 mM EDTA) for 2 minutes, washed and re-suspended in 1X PBS supplemented with 4% of fetal calf serum. Cell number, concentration and viability were tested by trypan blue dye exclusion test on Malassez Chamber and were later confirmed by flow cytometry analysis upon propidium iodide (1 mg/ml, Sigma) staining.

### Enrichment of pulmonary CD34^pos^ cells

Aliquots of 10^7^ dissociated cells were labeled using an indirect CD34 labeling technique with magnetic beads attached to a secondary antibody. Briefly, according to the manufacturer's instructions (Miltenyi Biotech), the cells were incubated with a 1:200 dilution of mouse anti-sheep CD34 antibodies (clone Eq8D11C1 kindly provided by Pr CD Porada, University of Nevada) for 15 min on ice, washed in a buffer containing 1X PBS, 2 mM EDTA and 0.5% bovine serum albumin. The mixture was then incubated for 15 min on ice with goat anti-mouse IgG microbeads (Miltenyi France) at a 1:5 dilution in 1X PBS, 2 mM EDTA, and 0.5% bovine serum albumin. The suspension was washed and centrifuged at 450 g for 10 min at 4°C. For the magnetic separation, Mini Macs separation columns (Miltenyi France) were rinsed with 500 μl of cold 1X PBS, 2 mM EDTA and 0.5% bovine serum albumin. The cell pellets were re-suspended at 10^7^ cells per 500 μl of the same buffer and poured into the column reservoir. The CD34 positive (CD34^pos^) cells were retained onto the magnetized matrix of the column, whereas the non-labeled cells passed through and were collected as the “non-retained” fraction. The columns were rinsed three times with 500 μl of 1X PBS, 2 mM EDTA and 0.5% bovine serum albumin. In order to collect the CD34^pos^ cells, the column were removed from the magnetic field and were washed by gravity with 1.5 ml of 1X PBS, 2 mM EDTA.

### Gene expression analysis of NANOG, BMI1 and OCT4 mRNAs by RT-PCR

Total RNAs were isolated using the RNeasy Mini kit (Qiagen, France) and 200 ng of total RNAs were reverse transcribed into cDNA using the iScript cDNA Synthesis kit (Bio-Rad, France). Specific primers for ovine GAPDH, BMI1, OCT4 and NANOG were designed as follows (from 5' to 3'): GAPDH FOR CCACCAACTGCTTGGCCCCC, GAPDH REV CCTCGGCCATCACGCCACAG, SP-C FOR GCAACGCCTGGCCCTGAGT, SP-C REV CATAATGTAGCAGCAGGTTC, CCSP FOR GTCACCCTGACTCTCTTCTG, CCSP REV CAGGGCTGAAAGGTTCCAGG, CD34 FOR GATTGCACTGGTCACCTCG, CD34 REV CTCCACGTAATAAGGGTCTTC, OCT4 FOR CAAGAACATGTGTAAGCTGC, OCT4 REV CGATACTCGTCCGCTTTCTC, NANOG-for GGCAGAAAAACAACTGGCCGAGGAA, NANOG REV CCCCACATGGGCAGGTTTCCAG, BMI1 FOR GCCACAACCATAATAGAATGTC and BMI1 REV CCCTGGAACTAATTTGTATAC. The PCR reactions were performed using 10 ng of cDNA with the KAPA SYBR FAST kit (Cliniscience, France) as recommended.

### *In vitro* cell cultures of total primary cells, bronchioloalveolar progenitors, AEC II and club cells

After tissue dissociation, 1.6 × 10^5^ cells were seeded per well in 6-well plates previously coated with 10 mg/ml of fibronectin (Sigma), 1 μg/ml of type I collagen (Sigma) and 5 mg/ml of type IV collagen (Sigma) in Quantum 286 medium (PAA, Austria) supplemented with 5 ng/ml of HGF (Hepatocyte Growth Factor; Abcys), 10 ng/ml of KGF (Keratinocyte Growth Factor; Abcys), penicillin and streptomycin (named "complete Q286 medium") as previously reported [22] and maintained for 2 to 3 passages.

To amplify the bronchioloalveolar precursors, 5 × 10^3^ CD34^pos^ cells were seeded per insert (membrane with pore size of 0,4 μm) placed in 24-well plates coated with fibronectin and type I and IV collagens in "complete Q286 medium" and maintained for 2 to 3 passages. These culture conditions with specific medium and extracellular matrix had been defined as the "maintenance conditions".

In order to induce their differentiation into AECII, 5 × 10^3^ CD34^pos^ cells per well were directly seeded in 24 well plates coated with fibronectin, and type I and IV collagens with "complete Q286 medium" and maintained in culture up to 3 to 4 passages. These culture conditions had been defined as the "AECII conditions". For their differentiation into club cells, 5 × 10^3^ CD34^pos^ cells per well were seeded in 24 well plates coated with only 10 mg/ml of fibronectin and maintained in Quantum 286 without additional KGF and HGF (then named "basic Q286 medium") for 1 passage. These culture conditions had been defined as the "Club cell conditions".

### Phenotypic analysis

The sorted cells were characterized for their expression of SP-C and CCSP by flow cytometry. After their elution from the columns, cells were rinsed in the 1X PBS and 4% fetal calf serum then centrifuged at 450 g 10 min at 4°C. Aliquots of 1.10^5^ cells were stained with a 1:600 dilution of a rabbit anti-human proSP-C polyclonal serum (Millipore, AB3786) and a 1:500 dilution of mouse anti-human CCSP monoclonal antibodies (Abnova H00007356-M01). After incubation at 4°C for 1 hour, cells were stained with goat anti-rabbit IgGs conjugated to Alexa 488 (InVitrogen) and/or goat anti-mouse IgGs (Whole molecule) conjugated to R-Phycoerythrin (Sigma) for 30 minutes, at 4°C in the dark. Stained cells were centrifuged at 1000 g for 2 min at 4°C then fixed with 2% paraformaldehyde before flow cytometry quantification on a Becton Dickson FACSCalibur™ Flow cytometer and analysis using the CellQuest Pro software. To avoid signal overflow a color compensation of 10 to 23% was applied. Populations of interest were gated regarding their physical properties (forward and size scatters) to eliminate dead cells and cell debris. Immunostaining using only the secondary antibody were used as controls to adjust the detection threshold for each measured wavelength.

Expression of CCSP and SP-C was analyzed on fixed cultured cells or frozen lung sections. After fixation with 4% paraformaldehyde, cells or tissue sections were permeabilized with 0.2% Triton X100, washed with 1X PBS and incubated 1 h, at room temperature with a 1:600 dilution of a rabbit anti-human proSP-C polyclonal serum or a 1:500 dilution mouse anti-human CCSP monoclonal antibodies. Cells were then washed and stained with goat anti-rabbit IgGs conjugated to Alexa 488 or anti-mouse IgGs conjugated to R-phycoerythrin for 30 minutes, at 4°C in the dark. The nuclei had been stained with DAPI. As negative controls, labeling was conducted without primary antibodies. The slides were analyzed on an AxioImager Microscope (Zeiss).

## Abbreviations

SP-C: Surfactant Protein-C; CCSP: Club Cell Secretory Protein; AECI: Alveolar Epithelial type I Cell; AECII: Alveolar Epithelial type II Cell; OCT-4: Octamer-binding Transcription factor 4; BMI1: B lymphoma Mo-MLV insertion region 1 homolog.

## Competing interests

The authors FA, AAR, SD, CD, BG, FG, VC and CL declare that they have no competing interests. The author JFM declares being consultant for LFB.

## Authors’ contributions

FA: conception, design, acquisition, analysis and interpretation of the data; drafting of the manuscript, writing the article, revising it for intellectual content and final approval. AAR: design, acquisition, analysis and interpretation of the data, first draft. SD, CD, BG and FG: Acquisition and analysis of the data and final approval of the article. VC: Contributed to the conception and design of the experiments, revising the manuscript and final approval. JFM: Contributed to the conception of the study; drafting of the manuscript and revising it for intellectual content and final approval. CL: Conception and design of the experiments, acquisition of the data, analysis and interpretation of the data, drafting of the manuscript, writing the article, final approval and revision for intellectual content. All authors read and approved the final manuscript.
